# Comparative and phylogenetic analysis of *Asparagus meioclados* Levl. and *Asparagus munitus* Wang et S. C. Chen plastomes and utility of plastomes mutational hotspots

**DOI:** 10.1038/s41598-023-42945-x

**Published:** 2023-09-20

**Authors:** Yulu Tian, Xue Liu, Yuanjiang Xu, Benxia Yu, Le Wang, Xianyou Qu

**Affiliations:** 1 School of Ethnic Medicine, Chengdu University of Traditional Chinese Medicine, No. 1166 Liutai Avenue, Wenjiang District, Chengdu, 611137 China; 2 Chongqing Academy of Chinese Materia Medica, 34 Nanshan Road, Huangjueya, Nanan District, Chongqing, 400065 China; 3 College of Life Science and Food Engineering, Chongqing Three Gorges University, 666 Tianxing Road, Wanzhou District, Chongqing, 404100 China

**Keywords:** Plant evolution, Genome, Sequencing

## Abstract

Tiandong is a vital traditional Chinese herbal medicine. It is derived from the tuber root of the *Asparagus cochinchinensis* according to the Pharmacopoeia of the people’s republic of China (2020 Edition). On account of the similar morphology, *Asparagus meioclados* and *Asparagus munitus* were used as Tian-Dong in southwest China. Chloroplast (cp) genomes are highly active genetic components of plants and play an extremely important role in improving the efficiency of the identification of plant species. To differentiate the medicinal plants belonging to the genus *Asparagus*, we sequenced and analyzed the complete plastomes (plastid genomes) of *A. meioclados* and *A. munitus* and obtained two plastomes whose length changed to 156,515 bp and 156,381 bp, respectively. A total of 111 unique genes have been detected in plastome, which included 78 protein-coding genes, 29 tRNA genes and 4 rRNA genes. In plastomes of *A. meioclados* and *A. munitus*, 14,685 and 14,987 codons were detected, among which 9942 and 10,207 had the relative synonymous codon usage (RSCU) values higher than 1, respectively. *A. meioclados* and *A. munitus* have 26 SSRs patterns, among which *A. meioclados* was 25 and *A. munitu*s 21. The average Ka/Ks value was 0.36, and positive selection was detected in genes of the photosynthetic system (*ndhF* and *rbcL*) in *Asparagus* species. To perform the comparative analysis of plastomes, the two newly sequenced plastomes of the *A. meioclados* and *A. munitus* species were compared with that of *A. cochinchinensis*, and 12 hotspots, including 5 coding regions and 7 inter-genomic regions, were identified. Based on the whole plastome of *Asparagus*, 2 divergent hotspots (*accD* and *rpl32-trnL-UAG*) and 1 international barcode fragment (*rbcL*) were screened, which may be used as particular molecular markers for the identification of *Asparagus* species. In addition, we determined the phylogenetic relationship between *A. meioclados* and *A. munitus* in the genus *Asparagus*. This study enriches our knowledge of the molecular evolutionary relationships of the *Asparagus* genus and provides treasured data records for species identification, molecular breeding, and evolutionary analysis of this genus.

## Introduction

*Asparagus* is a genus belonging to the family Liliaceae, which contains 300 species, and it spreads in temperate and tropical areas all over the world except America. There are 31 *Asparagus* species in China, including 29 wild species and 3 cultivated species^1^. Some have high medicinal value, such as *Asparagus cochinchinensis* (Lour.) Merr., *Asparagus meioclados* H. Lev., and *Asparagus munitus* F. T. Wang & S. C. Chen. Among *Asparagus* plant materials, only the dried root tuber of *A. cochinchinensis,* which is used as Tian-Dong has been indexed in the latest edition of the Chinese Pharmacopoeia in China^2^. Tiandong has been used in China for thousands of years with the prominent effects treating fever, cough and vomiting, sore throat, constipation, and other diseases. *A. meioclados*, a species closely related to *A. cochinchinensis*, has been used as the “Mo tonic" in Yi medicine to treat palpitation, anxiety, fatigue, whooping cough (pertussis), cough, chest pain, constipation, abdominal pain, and rheumatism in particular provinces such as Sichuan, Guizhou, and Yunnan^3^. *A. munitus*, another species also closely related to *A. cochinchinensis*, has been mainly distributed in the southwest of Sichuan (Muli) and northern Yunnan (Yongning)^4^. Because of the medication habits and preferences in a few regions of China, the indiscriminate use of *A. meioclados* and *A. munitus* as *A. cochinchinensis* has posed hidden risks to the accuracy and safety of clinical trials of Tiandon^5^, and been not conducive to the quality control of the traditional Chinese medicine Tiandong. Some phenotypic characteristics of *Asparagus* are overlap. Some phenotypic characteristics of *Asparagus* overlap. The medicinal plants of the genus *Asparagus* have branched leaves that are both diverse and variable in shape within species, and the base of the stem that produces scale-like leaves extends into stiff spines of different lengths. Moreover, the short flowering period and similar flower color make their identification difficult^6^. Therefore, the identification and classification of species in the genus *Asparagus* was controversial. To solve these problems, many scholars studied the phylogenetic relationships among a few species of the genus *Asparagus* based on morphology^7–9^, ribosomal DNA (rDNA) sequence^10,11^, and combination of a single or a few genes of chloroplast DNA (cpDNA)^12–18^, identified a few species and clarified the relationships among them, however, *A. meioclados* and *A. munitus* were not among these species. The identity of and phylogenetic relationships among the related *Asparagus* species remain completely unresolved.

Chloroplast (cp) is a substantial plant organelle with a prokaryotic origin that performs vital functions in the transfer and expression of genetic information in the life cycle of plants and algae^19^. At present, the plastomes of numerous plant species sequenced rapidly and efficiently, have been used in phylogenetic studies and for the identification of related species and have a higher copy number and relatively small size compared to the nuclear genomes. The plastomes are maternally inherited in angiosperms and highly conserved and have been proven effective in phylogenetic analysis for clarifying complex phylogenetic relationships^20–22^. Furthermore, chloroplast genome data are a useful aid to the development of plastid genetic markers in phylogenetic studies^23–25^.

To distinguish the medicinal plants in the genus *Asparagus* and determine the phylogenetic relationships among them, we sequenced the complete plastome of *A. meioclados* and *A. munitus* for the first time, conducted a comprehensive analysis of these plastomes to obtain useful super barcodes and specific DNA barcodes based on hypervariable fragment for the molecular identification of Tiandong, clarify the phylogenetic relationship of the genus and provided basic genetic information for further understanding the evolutionary relationship of *Asparagus*.

## Results

### Plastome sequencing and assembly

After DNA sequencing on the Illumina platform, 21,879,295 and 22,675,012 clean reads were obtained for A. meioclados and *A. munitus*, respectively. The average depth of *A. meioclados* and *A. munitus* were 1452 and 1952, respectively. The line plots (Figs. S1–2) based on the genome position information and coverage showed that all of the location of the genome had high coverage (no breakpoints), which proved that the assembly results of the plastids were reliable.

### Plastome structure and characterization in *A. meioclados *and *A. munitus*

**Figure 1:**
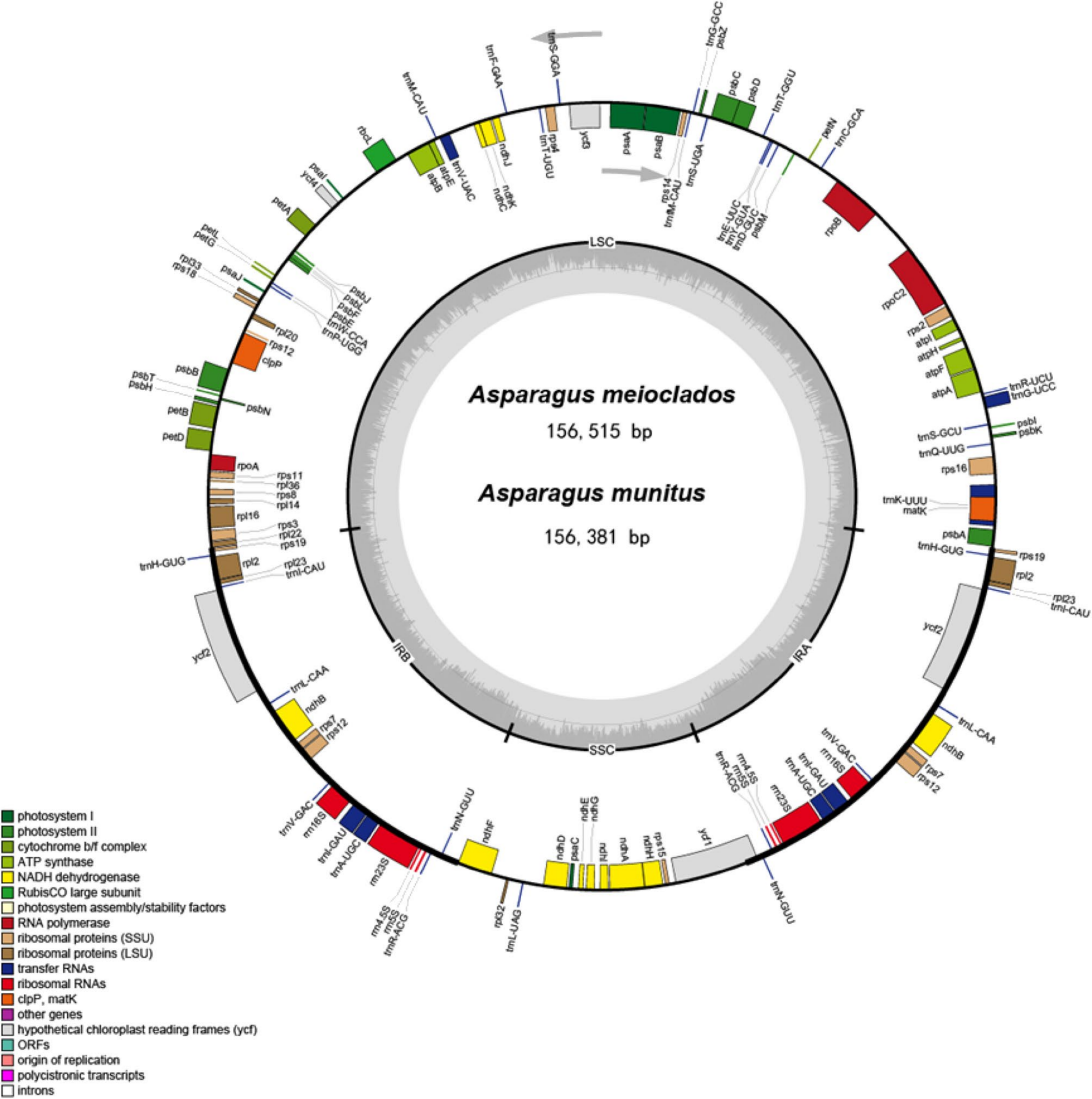
Plastome map of *A. meioclados* and *A. munitus*. The circle diagram showed all the genes in the small single copy (SSC) region, the large single copy (LSC) region, and the reverse repeat (IRa and IRb) region, with different functional groups of genes represented in different colors. Genes in the circle were transcribed clockwise and genes outside the circle were transcribed anticlockwise. The AT/GC contents correspond to the light and dark gray of the inner circle, respectively.

The plastomes of *A. meioclados* and *A. munitus* have the typical quadripartite structure consisting of two inverted repeat regions (IRA and IRB), a large single copy region (LSC) and a small single copy region (SSC). The difference in the size of the two plastomes was found to be 138 bp, which mostly occurred at the LSC (Fig. 1). In addition, 111 unique genes were encoded in each plastome, which included 78 protein-coding genes, 29 tRNA genes and 4 rRNA genes (Table 1). Moreover, there were a total of 19 genes located in the IR regions, including 7 protein-coding genes, 8 tRNAs, and 4 rRNA. Besides, the genes *ycf1* and *ndhF* were located at the junction of the SSC/IR borders, while *rpl22* was located at the LSC/IR border. A total of 16 genes, comprising 6 tRNAs (2 *TrnA-UGC*, 2 *trnI-GAU*, *trnK-UUU*, and *trnG-UCC*) and 10 protein-coding genes (*atpF*, *ndhA*, 2 *ndhB*, *petB*, *petD*, 2 *rpl2*, *rpl16*, and *rps16*), contained 1 intron. The genes *clpP*, *ycf3*, and *trnV-UAC*, however, contained 2 introns (Table S1). The guanine-cytosine (GC) content in IR regions was 42.91% and 42.92%, higher than that in other regions, in *A. meioclados* and *A. munitus*, respectively. Overall, the GC content in the IR, LSC, and SSC regions between the two species was similar.

**Table 1:** Features of two *Asparagus* plastomes.

Species	*A. meioclados*	*A. munitus*
Genome size (bp)	156,515	156,381
LSC (bp)	84,775	84,635
IRs (bp)	53,092	53,118
SSC (bp)	18,648	18,628
Total number of genes (unique)	111	111
Protein-coding gene (unique)	78	78
rRNA (unique)	4	4
tRNA (unique)	29	29
GC content (%)	37.55	37.57
LSC (%)	35.55	35.56
IR (%)	42.91	42.92
SSC (%)	31.40	31.43

### Codon usage bias analysis

**Figure 2:**
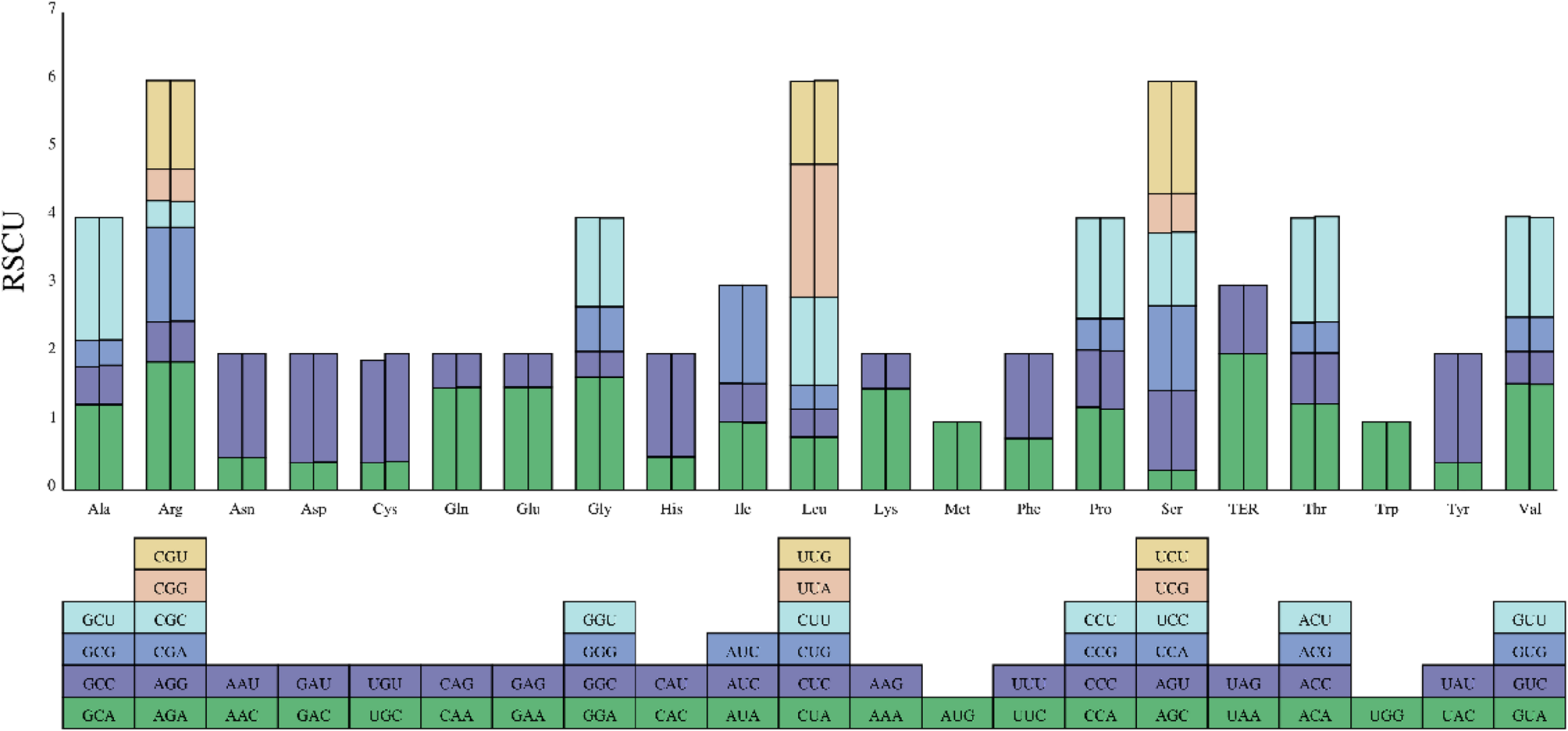
Codon content of CDS in the plastomes of *A. meioclados* (left) and *A. munitus* (right). The abscissa represented 20 amino acids and terminators, and the ordinate represented the RSCU value.

**Figure 3:**
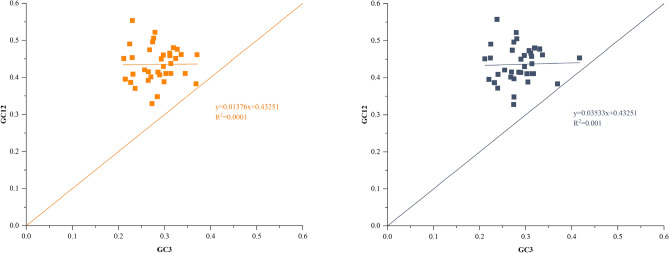
Neutrality analysis performed by plotting GC12 values against GC3 values for the plastomes of *A. meioclados* (orange) and *A. munitus* (blue). The diagonal line on the neutrality plot showed that the value of GC12 was equal to GC3.

To keep away from sampling errors in the analysis of codon usage bias, we estimated the relative synonymous codon usage frequency (RSCU) for 36 common eligible protein-coding sequences of *A. meioclados* and *A. munitus* (Fig. 2). The consequences showed that the codons of all 20 amino acids in the plastome of *A. meioclados* and *A. munitus* were identical, with a total of 36 protein-coding genes containing 14,685 and 14,987 codons, respectively, among which 9942, 10,207 had RSCU > 1 (Tables S2, S3). Leucine (Leu) and cysteine (Cys) were the most and least frequently used amino acids. All amino acids except Met and Trp were encoded by multiple synonymous codons, and Arg, Leu and Ser synonymous codons more than others. There were 14 amino acids with at least one codon which RSCU > 1, indicating that the codon is strongly preferred. In addition to the termination codon, the UUA codon of Leucine (Leu) had a RSCU value of 1.95, indicating its high frequency of use in the genome. Except for Met (Methionine-AUG) and Trp (Tryptophan-UGG), all of the other amino acid codons showed a strong bias towards A or U at the third position in codon. In addition, the EMBOSS software package was used to calculate the GC content at the first, second and third base in codon (GC1, GC2 and GC3, respectively) of the genome. Then, the GC3 and GC12 (mean values GC content of GC1 and GC2), were divided into horizontal and vertical coordinates to draw neutral plots to analyze the relative contribution of mutation pressure and natural selection to the formation of codon usage patterns (Fig. 3). The neutral map showed a weak correlation between GC3 and GC12, and the correlation coefficients for *A. meioclados* and *A. munitus* were 0.01376 and 0.03533, respectively. According to the neutrality plots, the slope of the regression line was close to 0, and all the genes represented by scatter points were on the diagonal line. The slope of the regression line for *A. meioclados* was lower than that for *A. munitus*, and the points fell almost on a horizontal line.

### Characterization of repeat sequences and simple sequence repeats (SSRs)

**Figure 4:**
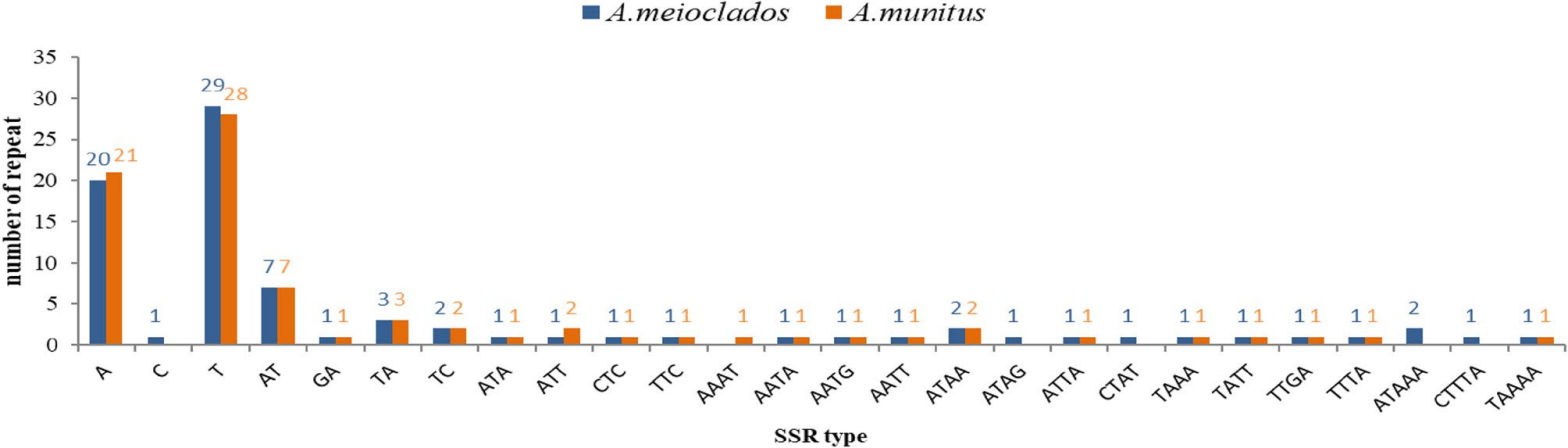
Repeat types, number and length of plastomes in *A. meioclados* and *A. munitus.*

**Figure 5:**
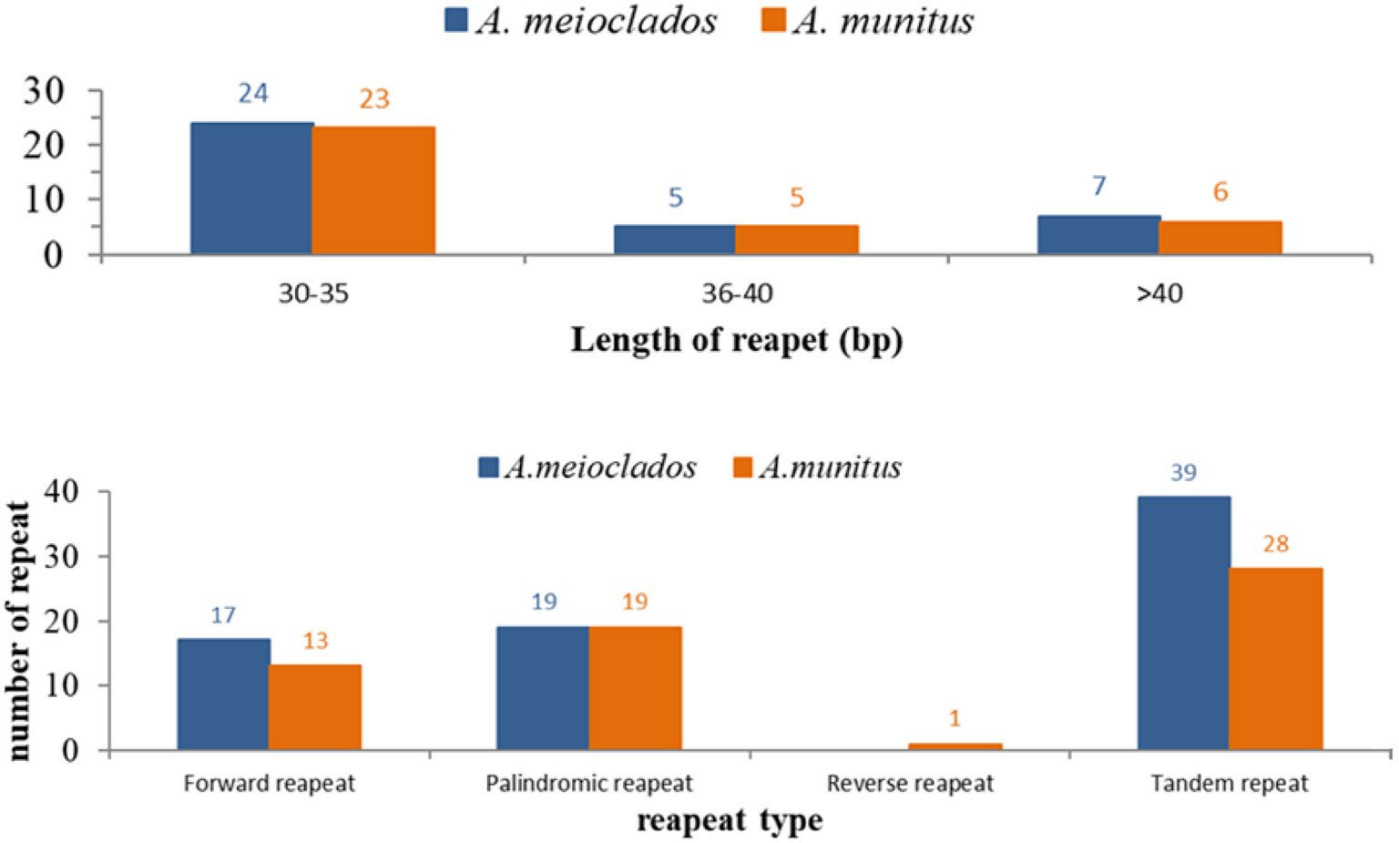
Frequency of repeat sequences of the *A. meioclados* and *A. munitus* plastomes determined by REPuter.

After scanning SSRs among the plastomes, a total of 79 and 83 SSRs in *A. meioclados* and *A. munitus* were detected, respectively, which comprised mono-, di-, tri-, and tetra-nucleotide repeats, but pentanucleotide repeats were only detected in *A. munitus* (Table 2). We observed a total of 26 SSRs patterns in two plastomes among which 25 in *A. meioclado*s and 21 in *A. munitus* (Fig. 4). Among the mononucleotide SSRs, polyadenine (polyA) or polythymidine (polyT) repeat patterns occurred most frequently, with only 1 cytosine (C) repeat and no guanine (G) repeat. Tandem repeats were the most frequently occurring repeats with 39 and 28 repeats detected in *A. meioclados* and *A. munitus*, respectively. Nineteen palindromic repeats were detected in both *A. meioclados* and *A. munitus*, 17 and 13 forward repeats were found, respectively. Only 1 reverse repeat was detected in *A. munitus*. The length of LSR in *A. meioclados* ranged from 30 to 52 bp, while for *A. munitus*, it was between 30 and 56 bp. The plastome of *A. meioclados* contained 24 repeats of 30–35 bp, 5 repeats of 36–40 bp, and 7 repeats longer than 40 bp. The plastome of *A. munitus*, however, contained 23 repeats of 30–35 bp, 5 repeats of 36–40 bp, and 6 repeats longer than 40 bp. Except for *accD* and *atpF* genes, most long sequence repeats (LSR) were located in non-coding regions (Fig. 5).

**Table 2:** Distribution of repeat number in plastomes in *A. meioclados* and *A. munitus*.

Type	*A. meioclados*	*A. munitus*
Simple sequence repeat (SSR)		
LSC	51	47
IR	12	12
SSC	20	20
Intron	7	4
Intergenic	47	52
Exon	29	23
Mono-nucleotide	50	49
Di-nucleotide	13	13
Tri-nucleotide	4	5
Tetra-nucleotide	12	11
Penta-nucleotide	4	1
Total	83	79
Tandem repeat		
LSC	26	17
IR	7	7
SSC	6	4
Intron	1	2
Intergenic	30	16
Exon	8	10
Total	39	28
Reverse repeat		
Forward repeat	17	13
Palindromic repeat	19	19
Reverse repeat	0	1
Total	36	33

### Divergent hotspots identification of three medicinal species

**Figure 6:**
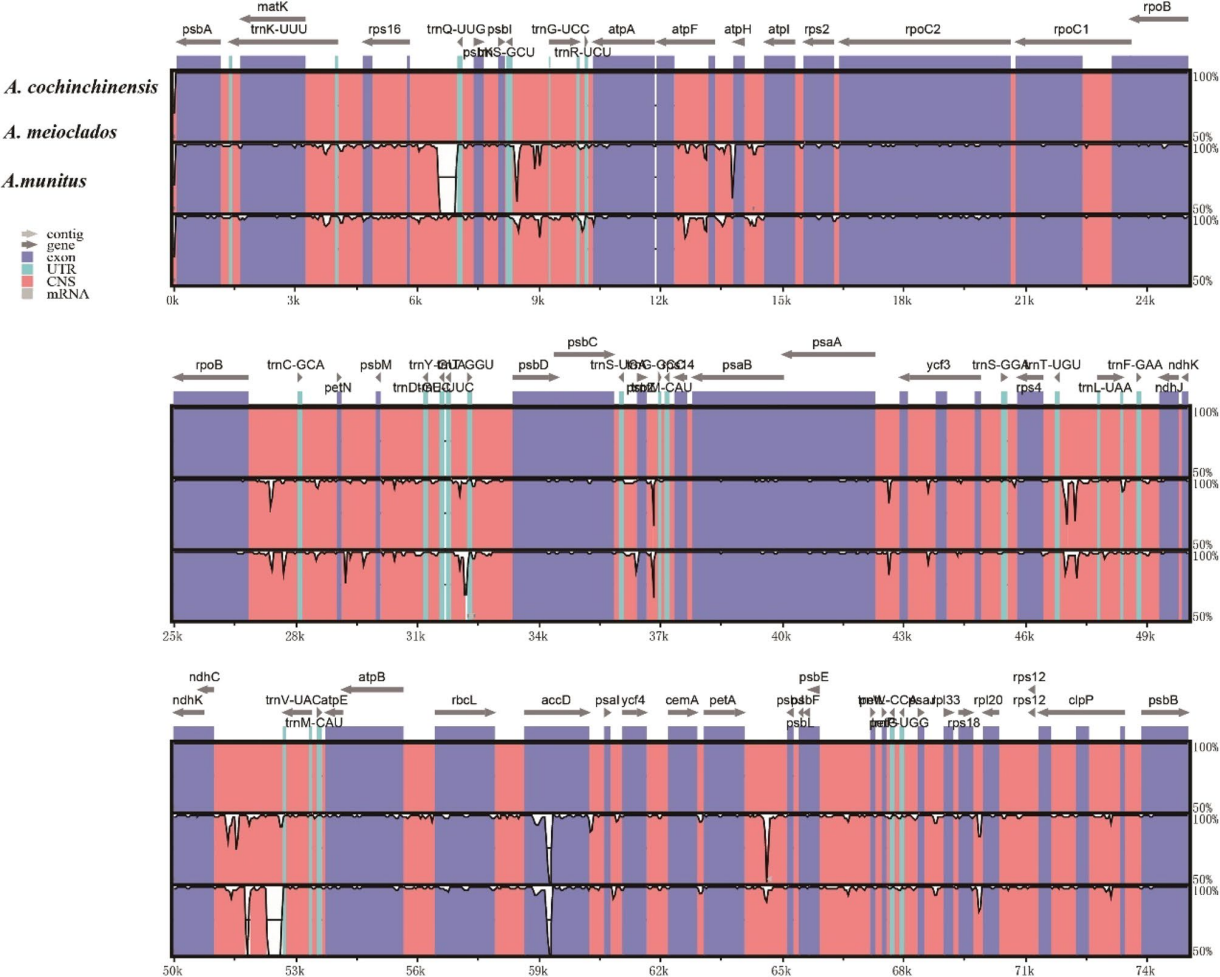
Comparison of three plastomes using mVISTA program with *A. cochinchinensis* plastome as a reference. The top gray arrows above the alignment indicate genes and their orientation. Genome regions are color coded. Blue and red areas indicate protein coding regions and the conserved non-coding sequences (CNS) regions, respectively. The green areas represent untranslated region (UTR) that do not encode proteins but have translational regulatory functions.

To detect the sequence polymorphisms that could be used as candidate sites in molecular markers, we performed mVISTA, IRscope, and sliding window analyses of the plastomes of *A. meioclados* and *A. munitus*, with *A. cochinchinensis* used as the reference. The results showed that the three plastomes were similar. Divergent hotspots mainly concentrated in the non-coding region and a few exonic regions, while the coding region was found to be more conserved. The sequence variations detected in LSC and SSC regions were greater than those in IR regions. There were 15 intergene spacer regions (*rps16-trnQ*, *trnS-trnG*, *atpF-atpH*, *rpoB-trnC*, *petN-psbM*, *trnE-trnT*, *psaA-ycf3*, *trnT-trnL*, *ndhC-trnV*, *petA-psbJ*, *rps18-rpI20*), *rpl16-rps3*, *rpl32-trnL*, *ccsA-ndhD*, and *rps15-ycf1* and 2 gene regions (*accD* and *ycf1*) had the highest difference (Fig. 6).

**Figure 7:**
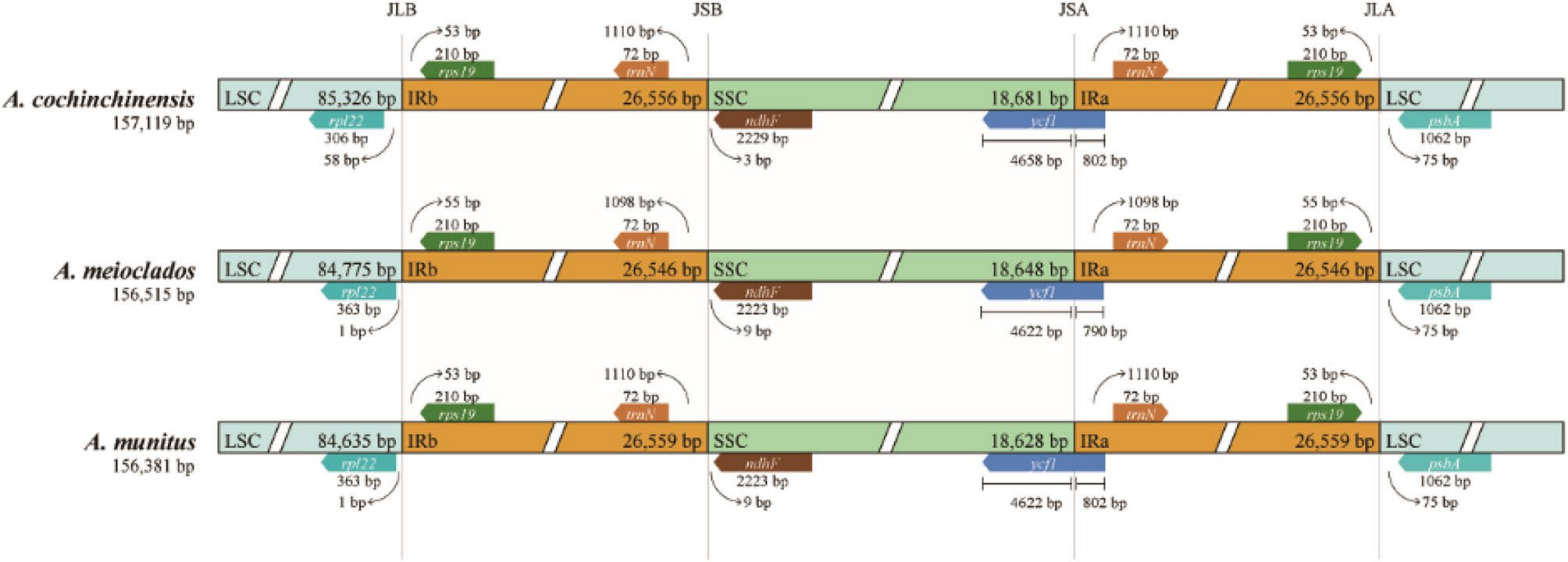
Boundary difference analysis of the quadripartite structure of three plastomes of the genus *Asparagus*.

The expansion and contraction of IR boundaries are the main reasons for differences in the size among cp genomes of plants during evolution^25^. The IRscope analysis showed that the gene types of LSC, SSC, IRa, and IRb joining areas were the same in the genome of the three species (Fig. 7), which had the characteristics of conservation. The genes of *rpl22*, *rps19*, *trnN*, and *ndhF*, exhibited differences in the location of the boundaries; the *rpl22* genes in *A. meioclados* and *A. munitus* were 2 bp away from the IRb-LSC border; while the *A. cochinchinensis* was 58 bp away from the IRb-LSC border. The *rps19* genes in *A. cochinchinensis* and *A. munitus* were located 53 bp away from the IRb-LSC border, whereas, in *A. meioclados,* they were 55 bp away from the IRb-LSC border. It is noteworthy that in these three plastomes, the *ycf1* gene existed in both the IRa and SSC regions, including the IRa-SSC junction. The length of IRa in the *ycf1* in *A. munitus* was 802 bp, while in *A. meioclados*, it was 790 bp.

**Figure 8:**
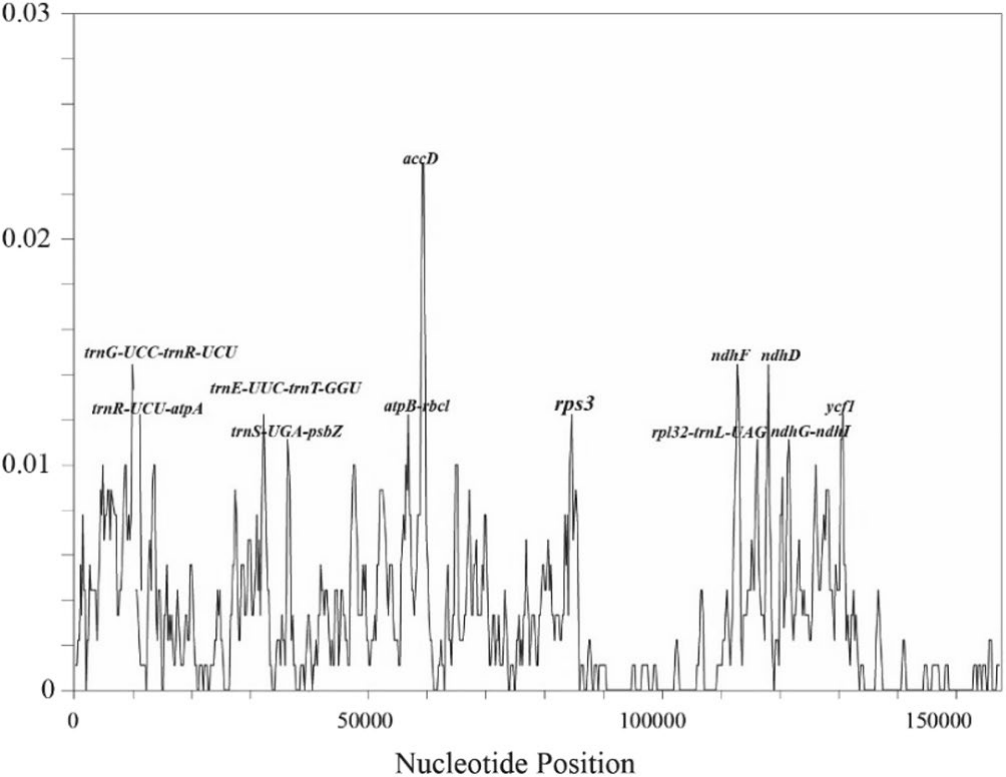
Nucleotide diversity (Pi) values among the three plastomes.

We compared the nucleotide diversity (Pi) values of the three plastomes, which were found to be within the range of 0–0.02333. We mapped 12 mutation hotspots (Fig. 8), which Pi > 0.01, including 5 coding regions (*accD*, *ndhF*, *ndhD*, *rps3*, and *ycf1*) and 7 intergenic regions (*ndhG-ndhI*, *rpl32-trnL-UAG*, *trnE-UUC-trnT-GGU*, *trnG-UCC-trnR-UCU*, *atpB-rbcl, trnR-UCU-atpA*, and t*rnS-UGA-psbZ*).

### Selective pressure analysis

**Figure 9:**
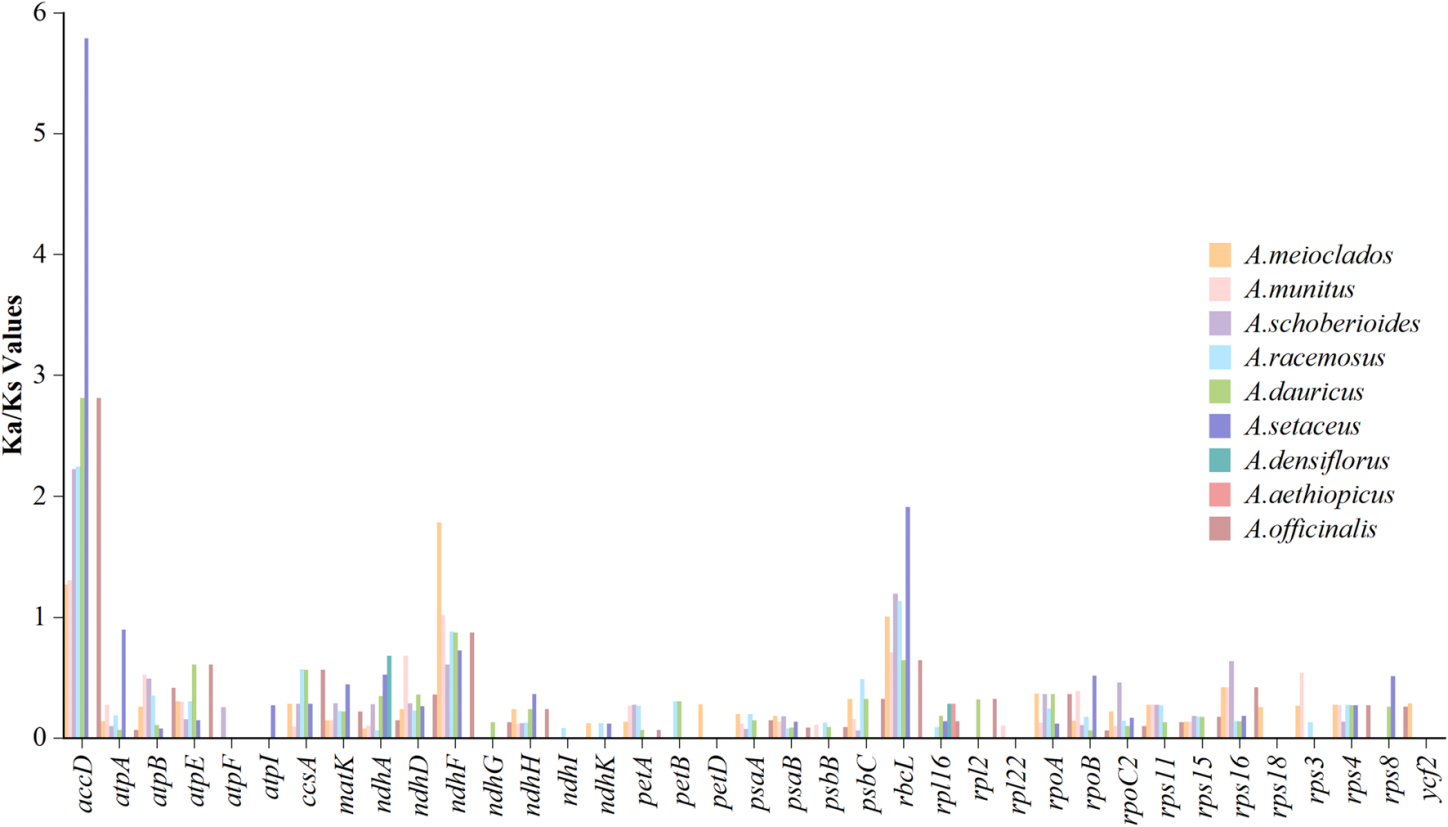
The Ka/Ks ratios of 37 protein-coding genes of *A. cochinchinensis* plastome when compared to other nine species of *Asparagus*.

The non-synonymous to synonymous substitution ratio (Ka/Ks) of 72 protein-encoding genes in 9 plastomes were compared with those of *A. cochinchinensis* to evaluate the selection pressure between species. The average Ka/Ks ratio of these genes was 0.36. Since the Ks value of 33 genes was 0, the rest (only 39 genes) were used in the Ka/Ks ratio test (Fig. 9). Using the KaKs calculator software, the Ka/Ks ratio of less than 0.5 was obtained for most genes, indicating that there were obvious patterns of purifying selection for these protein-encoding genes. A few photosynthesis-related genes showed positive selection in partially paired species (Ka/Ks ratio > 1), e.g., the *ndhF* gene was positively selected in 2 pairs (*A. cochinchinensis* & *A. meioclados* and *A. cochinchinensis* & *A. munitus*), *rbcL* gene was positively selected in 4 two-pair species (*A. cochinchinensis* & *A. meioclados*, *A. cochinchinensis* & *A. racemosus*, *A. cochinchinensis* & *A. setaceus* and *A. cochinchinensis* & *A. schoberioides*). This suggested that the need for adequate light in some species of *Asparagus* might have exerted a strong selective force on these genes during evolution.

The *accD* genes of all paired species except *A. cochinchinensis* & *A. aethiopicus* and *A. cochinchinensis* & *A. densiflorus* were positive selected, and the expression of *accD* genes might indirectly affect the efficiency of photosynthesis, possibly as a result of the genus's adaptation to its poor living environment.

### Phylogenetic analysis

**Figure 10:**
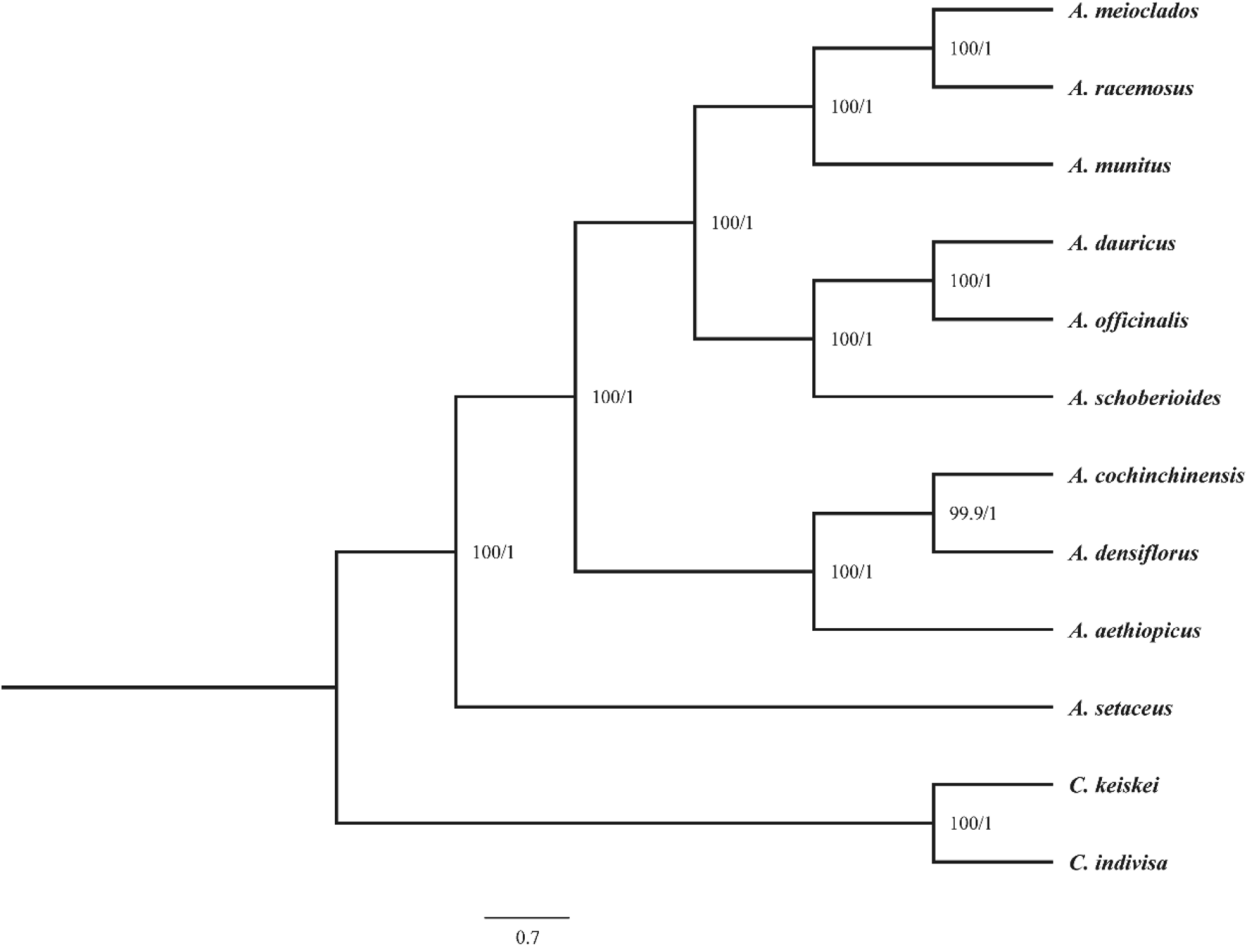
Phylogenetic tree reconstruction of the 10 species inferred from Bayesian inference (BI) and maximum likelihood (ML) based on cp DNA. Numbers represent ML/ BI posterior probabilities.

To avoid the heterogeneity of evolutionary rate between genes, misjudgment of orthologous lineages, and incomplete lineage sorting, which may affect the phylogenetic reconstruction, we used concatenation method (maximum likelihood and Bayesian inference) and coalescence method (wASTRAL) to reconstruct the phylogenetic relationship of *Asparagu*s and evaluated the phylogenetic positions of *A. meioclados* and *A. munitus*. Based on the maximum likelihood (ML), Bayesian inference (BI) analysis and wASTRAL of the entire plastomes, a consistent topology was obtained. The phylogenetic positions detected in this study were strongly supported by bootstrap values and posterior probabilities from ML, BI and wASTRAL analyses, respectively, which were annotated at corresponding nodes (Fig. 10, Fig S3). *Asparagus* species and the outgroup species were divided into different clades, e.g., 10 *Asparagus* species were grouped into four clades, which were sister clades, and *A. meioclados*, *A. munitus*, and *A. racemosus* were clustered into a single branch, while *A. dauricus*, *A. officinalis*, and *A. schoberioides* were gathered into a single branch. Furthermore, *A. cochinchinensis*, *A. densiflorus*, and *A. aethiopicus* were clustered together, but *A. setaceus* alone was in a separate branch.

### Evaluation of DNA barcoding

To find the genomic fragments that could identify *Asparagus* species, we evaluated 12 divergent hotspots and 3 international barcode fragments (*matK*, *rbcL*, and *trnH-psbA*) in the plastomes in terms of the degree of the variation in fragments and the applicability of DNA barcoding for species identification. The length of 14 fragments ranged from 130 to 5694 bp, with *trnR-UCU-atpA* and *ycf1* being the shortest and the longest fragments, respectively (Table 3).The proportion of variable sites ranged from 0.77% (*trnR-UCU-atpA*) to 41.33% (*trnH-psbA*), parsimony informative sites ranged from 0.32% (*matK*) to 3.08% (*ndhG-ndhI*), Singleton sites accounted for 0.00% (*trnR-UCU-atpA*)–40.00% (*trnH-psbA*), in which the variable sites with proportion > 2% were *rbcL*, *accD*, *rnH-psbA, ndhG-ndhI*, *rpl32-trnL-UAG*, *trnE-UCC-trnT-GGU*, *trnS-UGA-psbZ*. The NJ tree was constructed using 12 divergent hotspots and 3 international common barcode fragments, and the identification rate of 10 *Asparagus* species was 30% (*trnR-UCU-atpA*)–100% (*atpB-rbcL*, *ndhF*, and *accD*), among them, the identification rate > 80% were *accD*, *ndhF*, *rbcL*, *ycf1*, *atpB-rbcL*, and *rpl32-trnL-UAG* (Table S4). Finally, 2 divergent hotspots (*accD* and *rpl32-trnL-UAG*) and 1 international barcode fragment *(rbcL*) were selected, which identification rate greater than 80% and the proportion of variable sites greater than 2%. These divergent hotspots could be used as specific DNA barcodes the identification of *Asparagus* species. The Neighbor Joining (NJ) tree (Figs. S4–S6) based on divergent hotspots showed highly similar topological relationships to the plastome ML tree, with only a few differences in *A. cochinchinensis*, *A. densiflorus* and *A. aethiopicus*. *A. cochinchinensis* was sister to *A. densiflorus* and *A. aethiopicus* in the ML tree of the plastome. However, the neighbor-joining (NJ) tree results based on *accD* showed that *A. cochinchinensis*, *A. densiflorus* and *A. aethiopicus* were one clade. The phylogenetic relationship obtained by Neighbor Joining (NJ) tree of *Asparagus* based on *rpl32-trnL-UAG* was consistent with that obtained by *accD*. The phylogenetic relationship obtained by Neighbor Joining (NJ) tree of *Asparagus* based on *rbcL* was completely consistent with that obtained by plastome ML tree. Except for the *A. aethiopicus* and *A. cochinchinensis* branches (Bootstrap value = 0.31), the other branches were given high support (Bootstrap value > 0.5).

**Table 3:** Sequence diversity comparison of 12 divergent hotspots and 2 international barcode fragments in 10 *Asparagus* species.

Gene	Aligned length	Variable sites	Parsimony informative sites	Singleton sites	% of variable sites	% of parsimony informative sites	% of Singleton sites
*matK*	1563	18	5	13	1.15	0.32	0.83
*rbcL*	1443	33	20	13	2.29	1.39	0.90
*trnH-psbA*	75	31	1	30	41.33	1.33	40.00
*accD*	1220	54	25	29	4.43	2.05	2.38
*ndhD*	1245	18	9	9	1.45	0.72	0.72
*ndhF*	2223	40	19	21	1.80	0.85	0.94
*rps3*	687	8	3	5	1.16	0.44	0.73
*ycf1*	5694	104	75	29	1.83	1.32	0.51
*atpB-rbcL*	812	14	4	10	1.72	0.49	1.23
*ndhG-ndhI*	390	20	12	8	5.13	3.08	2.05
*rpl32-trnL-UAG*	1020	29	13	16	2.84	1.27	1.57
*trnE-UCC-trnT-GGU*	508	19	12	7	3.74	2.36	1.38
*trnG-UCC-trnR-UCU*	141	8	3	5	5.67	2.13	3.55
*trnR-UCU-atpA*	130	1	1	0	0.77	0.77	0.00
*trnS-UGA-psbZ*	385	19	9	10	4.94	2.34	2.60

## Discussion

Similar to most angiosperm plastid genomes^25^, the plastomes of *A. meioclados* and *A. munitus* exhibited a typical quadripartite structure consisting of 2 reverse repeat regions (IRA and IRB), a large single copy region (LSC) and a small single copy region (SSC). The plastomes of *A. meioclados* and *A. munitus* were slightly smaller than those of other *Asparagus* species^26,27^. The length of LSC, IR and SSC regions, gene content (Table 1), gene order, and number of introns for *A. meioclados* and *A. munitus* showed high similarity, similar with plants of the genus *Camellia*^28^, suggesting that the chloroplast genome was relatively conserved. The variation in the frequency of synonymous codons used in protein synthesis is of great significance for studying the origin and evolution of species, gene functions, and protein expression^29,30,73^. The codons encoding all amino acids, except for Met and Trp, showed a strong bias for A or U in the third codon positions. It was in agreement with *Cicer echinospermum*^31^ and *Carpesium abrotanoides*^32^, but not with *Glechoma longituba*^33^ and *Aconitum coreanum*^34^. The difference might be related to the different number of tRNAs in different species, and the use of codons with high abundance of paired tRNAs could guarantee efficient protein expression. The neutral map showed a weak correlation between GC3 and GC12, indicating that the GC content of the genome displayed a conservative pattern, and the codon usage bias was mainly affected by selection. In addition, the slope of the regression curve of *A. meioclados* and *A. munitus* was 0.01376 and 0.03533, respectively, indicating that natural selection had an important effect on the formation of codon bias in *A. meioclados* and *A. munitus*.

The intra-species variations in simple sequence repeats (SSRs) could provide a valuable resource for population genetics of polymorphism^35,36^. Similar to other studies, the nucleotide repeats consisting of A or T had the highest number in *A. meioclados* and *A. munitus*, and the SSRs were mainly found in the non-coding region and large and small single-copy regions (LSC and SSC) in our study^18^.

Mononucleotide repeats were found to contribute more to genetic variations than other SSRs. Notably, most of the dinucleotide SSRs of *A. meioclados* and *A. munitus* were AT/TA/TC, which was consistented with previous reports of other angiosperms^37^. This confirmed that the simple repetitive sequence of chloroplast genome mainly consists of short poly real A and poly real T, rather than C or G^38^. Tandem repeated sequences have played a crucial role in genome size changes, genome rearrangement, gene duplication, evolution, and inheritance, and genetic variation^39,40^. Three types of tandem repeats were detected in *A. meioclados* and *A. munitus*, with the palindromic repeats being the most abundant repeats, followed by forward repeats. Only the plastome of *A. munitus* had inverted repeats, similar to the case observed for Chinese Polyspora^41^. These differences suggest significant sequence variation and genome rearrangements that have occurred during evolution.

Although the cp genomes of plants are conserved in size and structure, IR amplification and contraction are common evolutionary phenomena^42^. Comparative analysis of IR boundaries clearly showed differences in chloroplast genomes among *Asparagus* species. The results of mVISTA, IRscope, and sliding window analyses of the plastomes of *A. meioclados* and *A. munitus* confirmed that the differences in plastomes in the LSC region were greater than those in the SSC region and also greater in the non-coding region than in the coding region, possibly as a result of copy number differences in the inverted repeats sequences caused by gene conversion^43^. The results of the present study demonstrated that the differences in the nucleotide diversity greater than 1.5% in hotspots could be used for phylogenetic analysis, genetic analysis, and identification of plant seed species^44,45^. The identified hotspots included 7 intergenic regions (*ndhG-ndhI*, *rpl32-trnL-UAG*, *trnE-UUC-trnT-GGU*, *trnG-UCC-trnR-UCU*, *atpB-rbcl*, trnR-UCU-atpA, and *trnS-UGA-psbZ*), which could serve as potential molecular markers for identifying the three *Asparagus* species.

Adaptive evolution is defined as the improvement in the fitness of a plant species during evolution and has always been driven by evolutionary processes, like natural selection, that act to increase genetic variation sourced from mutation, genetic recombination, and gene flow and also contribute to biodiversity at all levels of biological organization^46^. In *Asparagus*, the *ndhF* and *rbcL* genes, which belong to the photosynthetic system, were found to be under positive selection. The *ndh* gene encodes cp NADH dehydrogenase^47^. The NADH dehydrogenase complex in higher plants is not only involved in photosynthetic electron transport but also resistant to photooxidative stress^48,49^. The *rbcL* gene encodes ribulose 1,5-bisphosphate carboxylase/oxygenase, and *rbcL* plays an important role as a regulator of photosynthetic electron transport and is required for photosynthesis^50^. This suggests that the need for enough light that some *Asparagus* species have might have exerted strong selective forces on these genes during evolution. The *accD* gene encodes the β-carboxyltransferase subunit of the acetyl-Coa carboxylase complex^51^ and plays an important role in leaf development and affects leaf lifespan and seed yield^52^. Additionally, the positive expression of the *accD* gene might indirectly affect the efficiency of photosynthesis, which could be due to the adaptation of the species of this genus to poor environments in which they grow. In summary, positive selection would possibly contribute to *Asparagus* species diversification and adaptation.

Numerous practical studies have demonstrated that organelle phylogenies are an important tool for clarifying the genetic relationships between species^46,53^. In the past two decades, there are a large variety of researches on biological networks emerging, which have attracted great attention from scientists and scholars^54^. In order to get more accurate phylogenetic relationship of the genus asparagus, we compared the concatenate method and coalescent method, obtained a consistent topology. The 10 species of *Asparagus* were divided into four evolutionary clades, *A. meioclados*, *A. munitus* and *A. racemosus* formed a single branch, while *A. dauricus*, *A. officinalis* and *A. schoberioides* gathered into another branch. *A. cochinchinensis*, *A. densiflorus*, and *A. aethiopicus* were grouped in a third branch, and *A. setaceus* was located in the most basic branch, which was only including its own. These results are consistent with the phylogenetic relationships reported in previous studies^18,55^, and further clarify the phylogenetic relationships within *Asparagus* species. According to the phylogenetic position of the species within the genus *Asparagus* determined in this study, *A. meioclados* was most closely related to *A. racemosus*, and *A. cochinchinensis* was closest to *A. densiflorus*. According to the traditional classification of *Asparagus*^56^, dioecious and monoecious species were divided into two major factions, but *A. setaceus* and *A. densiflorus* (both dioecious species) were in different branches with high bootstrap values in our study. The results showed that at the molecular level, monoecious species of the genus *Asparagus* were not necessarily completely divergent, more data might be needed to confirm this speculation.

In recent years, DNA barcoding has played an extremely important role in improving the efficiency of plant species identification. From single-locus DNA barcodes to combined markers and even to the genome level, DNA barcoding has provided more and more genetic information. The plastome has the potential to serve as a highly accurate "super DNA barcode" used for taxonomic revision, phylogeny inference, and species identification^57,58^. But the complete plastome sequencing is cost and time-consuming. A short, easily amplified, and variable sequence as an ideal DNA barcoding is becoming increasingly necessary for the study of Traditional Chinese Medicine^59^, and it has been successfully used for many taxa. The development of specific barcodes has been shown to make the rapid and accurate identification of specific taxa feasible^60–63^. However, due to the complex evolutionary history of plants, some groups cannot be identified using the existing international common barcodes (*matK*, *rbcL*, and *trnH-psbA*)^64,65^. Therefore, the development of new DNA barcodes with high identification rates and group specificity is currently being researched. In this study, 2 divergent hotspots (*accD*, and *rpl32*-*trnL*-*UAG*) and 1 international barcode fragment (*rbcL*) were identified from the whole plastomes of *Asparagus* species, with the discrimination rates higher than 80% and proportions of variable sites greater than 2%. These potential molecular markers could be used for identifying medicinal species of the genus *Asparagus*. In addition, other analysis and application of the chloroplast genome of *Asparagus*, such as SNP loci and digital PCR analysis techniques, could be used to further accurately identify *Asparagus* species.

## Conclusion

In this study, for the first time, the plastomes of *A. meioclados* and *A. munitus* were obtained. The results of genome sequencing, assembly, and annotation, as well as comparative analyses showed that both plastomes had similar gene and GC contents but differed in tandem repeats and SSRs. Twelve mutation hotspots, including 5 coding regions and 7 intergenic regions, were detected in the two plastomes compared with that of *A. cochinchinensis*. Phylogenetic analysis showed that *A. meioclados* and *A. munitus* were in different clades from *A. cochinchinensis*, and a more accurate assessment of phylogenetic relationships in *Asparagus* was carried out in the present work compared to previous studies. Two divergent hotspots and 1 international barcode fragment were selected as specific DNA barcodes for the identification of *Asparagus* species with a discrimination rate higher than 80% and a proportion of variable sites greater than 2%. With the development of plant science, plastid transformation is becoming an important tool for plant species identification. This study used chloroplast genome fragments for the molecular identification of *Asparagus* species and it can provide valuable information about the genetic diversity and evolutionary patterns of *Asparagus* species.

## Materials and methods

### Plant material, DNA extraction and sequencing

Fresh and healthy leaves of *A. meioclados* and *A. munitus* used in this study were collected from Miyi and Muli counties, Sichuan Province, respectively, and then they were frozen at − 80 °C. The tissue of all plant samples was used for the extraction of DNA with the modified cetyltrimethylammonium bromide (CTAB) method^66^ and the concentration and quality of DNA were determined by the Qubit 3.0 Fluorometer (Invitrogen) and agarose gel electrophoresis (1%), respectively. High-quality DNA samples (concentration ≥ 5 ng/μL, total yield higher than or equal to 0.5 µg, integrity of the main dispersion band above 5 kb, and no obvious impurities below the qualification threshold) were used for library construction and then sequenced by the Illumina NovaSeq 6000 (San Diego, CA, United States). Eight *Asparagus* plastomes for the comparison with the main 3 plastomes used in this study, including *A. cochinchinensis* (MW970105), *A. schoberioides* (KX790361), *A. officinalis* (NC_034777), *A. dauricus* (MT712151), *A. racemosus* (MN736960), *A. setaceus* (MT712152), *A. densiflorus* (MT740250), and *A. aethiopicus* (MZ337394), were downloaded from the NCBI GenBank.

### Plastome assemblage and annotation

After DNA sequencing on the Illumina platform, clean reads were obtained. Raw reads with a low average quality (the Phred quality score (Q) ≤ 20) were removed using Trimmomatic v 0.3^67^ with the parameters set as follows: sliding window 4–15, trailing 3, leading 3, and the minimum length of reads 50. The coverage depth of each site was counted using SAMTOOLS^68^, and the assembly quality of plastids was judged by drawing line plots based on genomic position information and coverage^69^. The plastomes were assembled de novo by GetOrganelle^70^. The online tool CPGAVAS2 (http://47.96.249.172:16019/analyzer/home)^71^, an integrated plastome sequence annotator and analyzer, was used to annotate the plastid genomes of *A. meioclados* and *A. munitus*, with *A. officinalis* (MT712156) and *A. schoberioides* (KX790361) used as references, respectively. The OGDRAW tool (https://chlorobox.mpimp-golm.mpg.de/OGDraw.html) with the default settings was used to draw the circular genome maps and then manually check them^72^. Finally, the annotated sequences for *A. munitus* and *A. meioclados* were submitted to GenBank (accession numbers OQ628361 and OQ628362, respectively).

### Codon usage bias

The variation in the frequency of synonymous codons used in protein synthesis is of great significance for the studies on the origin and evolution of species, gene functions, and protein expression^73^. To estimate the relative synonymous codon usage (RSCU) more accurately, all coding sequences (CDSs) were screened according to the following conditions: (1) deletion of repeated sequences; (2) removing CDSs shorter than 300 bp; (3) using ATG as the initiation codon; and (4) sequencing with termination codons (TAA, TAG, and ATG)^74,75^. CodonW v.1.4.2^76^ was used to calculate the RSCU value for qualified sequences to quantify the degree of codon use bias. The GC content was calculated by the EMBOSS software suite^77^. The neutrality plot analysis was performed to determine the relative contributions of mutation pressure and natural selection to the formation of codon usage patterns.

### Characterization of repeat sequences and SSRs

SSRs and Tandem repeat sequences of cp genome are important for genome structure, phylogenetic relationship and population genetic analysis^78^. Repeats in the newly sequenced plastomes of the two species, including palindromic, reverse, and direct repeats were identified using REPuter^79^ with the following settings: (1) a hamming distance of 3; (2) a minimum repeat size of 30 bp; and (3) maximum computed repeats of 5000. The simple sequence repeats (SSRs) of the plastomes of *A. meioclados* and *A. munitus* were identified using the MISA Perl script^80^ with the search parameters set at 10, 5, 4, 3, 3, and 3 for mono-, di-, tri-, tetra-, penta-, and hexanucleotide repeats, respectively. Tandem repeats were detected by the online tool Tandem Repeats Finder (https://tandem.bu.edu/trf/trf.html) with default parameter settings^81^.

### Genome comparison and divergent hotspots identification

To further develop molecular markers for the identification of medicinal plants of the genus *Asparagus*, we compared the newly sequenced plastomes of the two species with that of *A. cochinchinensis*. We used the mVISTA v.2.0^82^ program in the Shuffle-LAGAN mode to compare the plastomes. On line program Irscope^83^ (https://irscope.shinyapps.io/irapp/) was conducted to compare the large single copy (LSC), small single copy (SSC) and reverse repeat (IR) regions in plastomes. Finally, we identified the polymorphic regions. MAFFT^84^ was used to perform the multiple sequence alignment (MSA) analysis of plastomes, and the DnaSP v6^85^ software was used for performing the sliding window analysis. The nucleotide variability (Pi) of the coding and non-coding regions was calculated, and the window length and the step size were set to 600 and 200 bp, respectively.

### Selective pressure analysis

The ratio of the nonsynonymous substitution rate (Ka) to the synonymous substitution rate (Ks) (Ka/Ks) of protein-coding genes was used to explore the relationship between the growing environment and the evolutionary rates of protein-coding genes in the genus *Asparagus*. The Ka/Ks ratios of 10 species of the *Asparagus* genus were calculated using the YN model in KaKs_Calculator 2.0^86^, Since the YN model considers the characteristics of DNA sequence evolution, it has been increasingly applied in the study of molecular evolution^87,88^.

### Phylogenetic analysis

The whole chloroplast genomes of the above mentioned 8 species were used for phylogenetic analysis to determine the phylogenetic position of *A. meioclados* and *A. munitus* in the genus *Asparagus*, with *Convallaria keiskei* (accession number, NC_042228.1) and *Cordyline indivisa* (accession number, NC_035998) used as outgroups, respectively. The plastomes of these species were aligned using MAFFT^84^, and the best-fitting model of nucleotide substitution (GTR + I + G) was determined using the Akaike Information Criterion (AIC) in jModelTest V2.1.10^89^, MrBayes v3.2.6^90,91^, and IQ-TREE v2.1.4^92^ were use to perform phylogenetic analysis using Bayesian inference (BI) and maximum likelihood (ML) methods, respectively. The former used the Markov chain Monte Carlo (MCMC) algorithm and ran four chains for 1,000,000 generations at the same time, sampling every 1,000,000 generations. The first 25% of trees were discarded as burn-in. The remaining trees were used to generate a consensus tree, which was considered to reach a plateau, as the mean standard deviation (SD) of the split frequencies remained below 0.001. The latter [maximum likelihood (ML)] was calculated using ModelFinder in the IQ-TREE package and the Akaike Information Criterion (AIC) with 1000 bootstrap copies^93^. In addition, we reconstructed the species tree using two coalescence-aware methods: CASTER and wASTRAL. The aligned genomes were split into 340 segments of 500 base-pairs, and we estimated a gene tree for each segment by IQ-Tree with approximate Bayesian supports. The best nucleotide substitution model for each gene tree were find by IQ-Tree. CASTER-site v1.15.0.0^94^ was used to infer the species tree directly from the aligned genomes. Local bootstrap supports were assessed based on blocks of 100 base-pairs. We also inferred the species tree from estimated gene trees using wASTRAL-hybrid v1.15.2.3^95^ which utilized both branch lengths and branch supports. The built-in local posterior probabilities were displayed as branch supports. The phylogenetic tree visualized using FigTree v1.4 (http://tree.bio.ed.ac.uk/software/fgtree/).

### Evaluation of DNA barcoding

The divergent hotspots (pi > 0.015) and the international common barcode fragments (*matK*, *rbcL,* and *trnH-psbA*) in the plastomes of species were evaluated in terms of the degree of fragment variation and the efficiency of species identification. The MEGA v.11^96^ software was used to measure the proportion of variable sites, parsimony informative sites and singleton sites. Based on the K2P model, MEGA v.11 was used to construct the neighbor-joining (NJ) tree, and the bootstrap method was used to repeat the operation 1000 times to test the reliability (support value) of each branch. The success of sequence analysis was judged by the support value of species clustering (higher than 0.5). The number of fragments was counted to screen for suitable fragments as ideal DNA barcodes for the genus *Asparagus*.

### Ethical approval

The collection of plant materials (*A. meioclados* and *A. munitus*) used in this study complied with relevant institutional, national and international guidelines and legislation.

### Supplementary Information

Supplementary Figures.

Supplementary Tables.

## Data Availability

The plastome sequence of *A. munitus* and *A. meioclados* were submitted on the National Center for Biotechnology Information (NCBI), and the accession number were (OQ628361, OQ628362). (https://www.ncbi.nlm.nih.gov/nuccore/ OQ628361). (https://www.ncbi.nlm.nih.gov/nuccore/ OQ628362).
