# Supplementary material for: Comparative and phylogenetic analysis of Asparagus meioclados Levl. and Asparagus munitus Wang et S. C. Chen plastomes and utility of plastomes mutational hotspots
Source: Sci Rep. 2023 Sep 20;13:15622. doi: 10.1038/s41598-023-42945-x (PMC10511529; doi:10.1038/s41598-023-42945-x)
Supplement: Supplementary file 1 — Supplementary Figures. [file 41598_2023_42945_MOESM1_ESM.pdf]

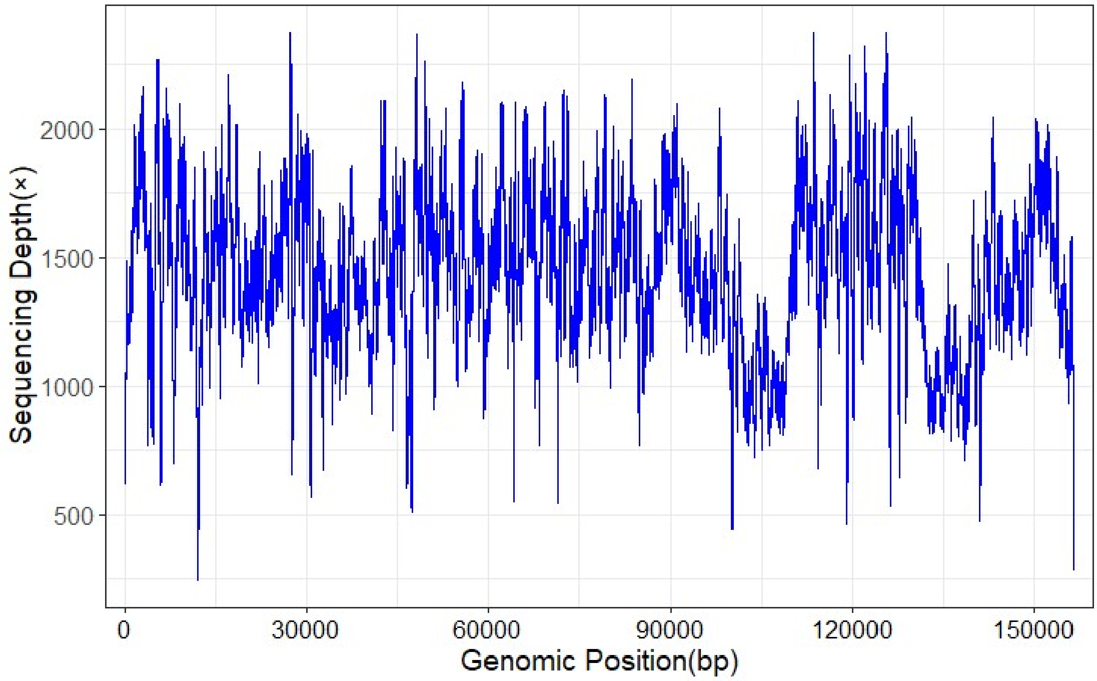

Supplementary figure 1. Line plots of chloroplast genome coverage in *A. meioclados*.

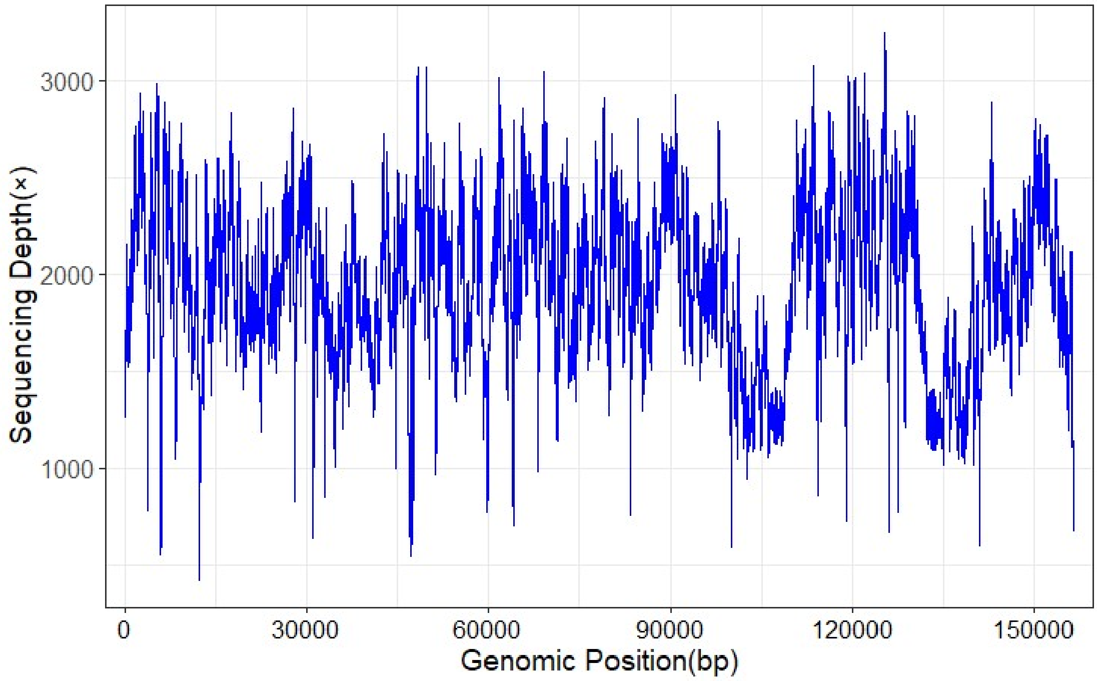

Supplementary figure 2. Line plots of chloroplast genome coverage in *A. munitus*.

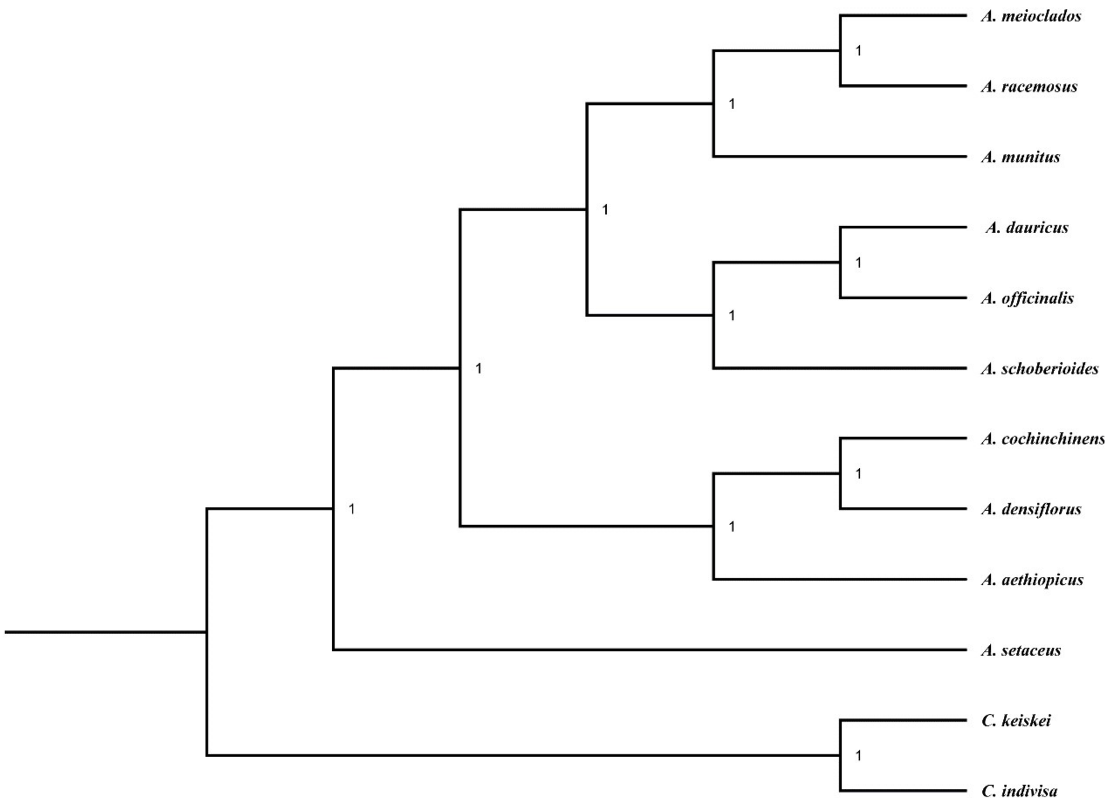

Supplementary figure 3. Phylogenetic tree of the 10 species inferred from wASTRAL analyses based on cp DNA. Numbers represent local posterior probabilities.

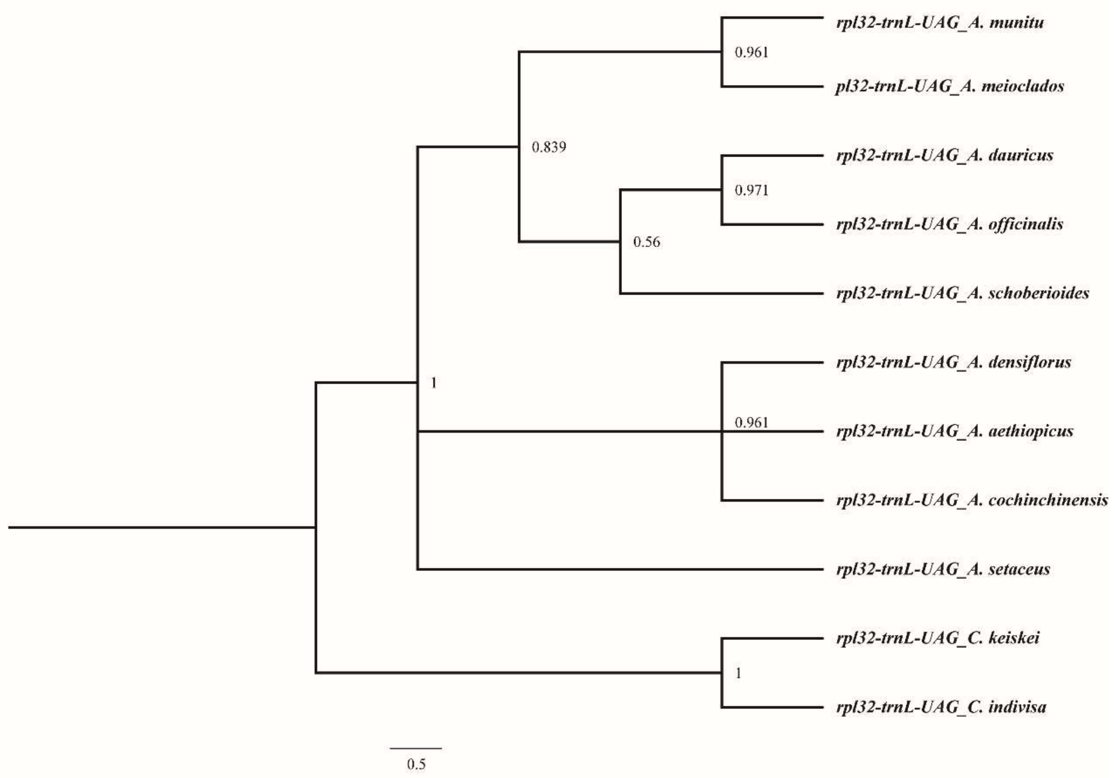

Supplementary figure 4. Neighbor Joining（NJ）tree of *Asparagus* based on *accD*, numbers represent Bootstrap values.

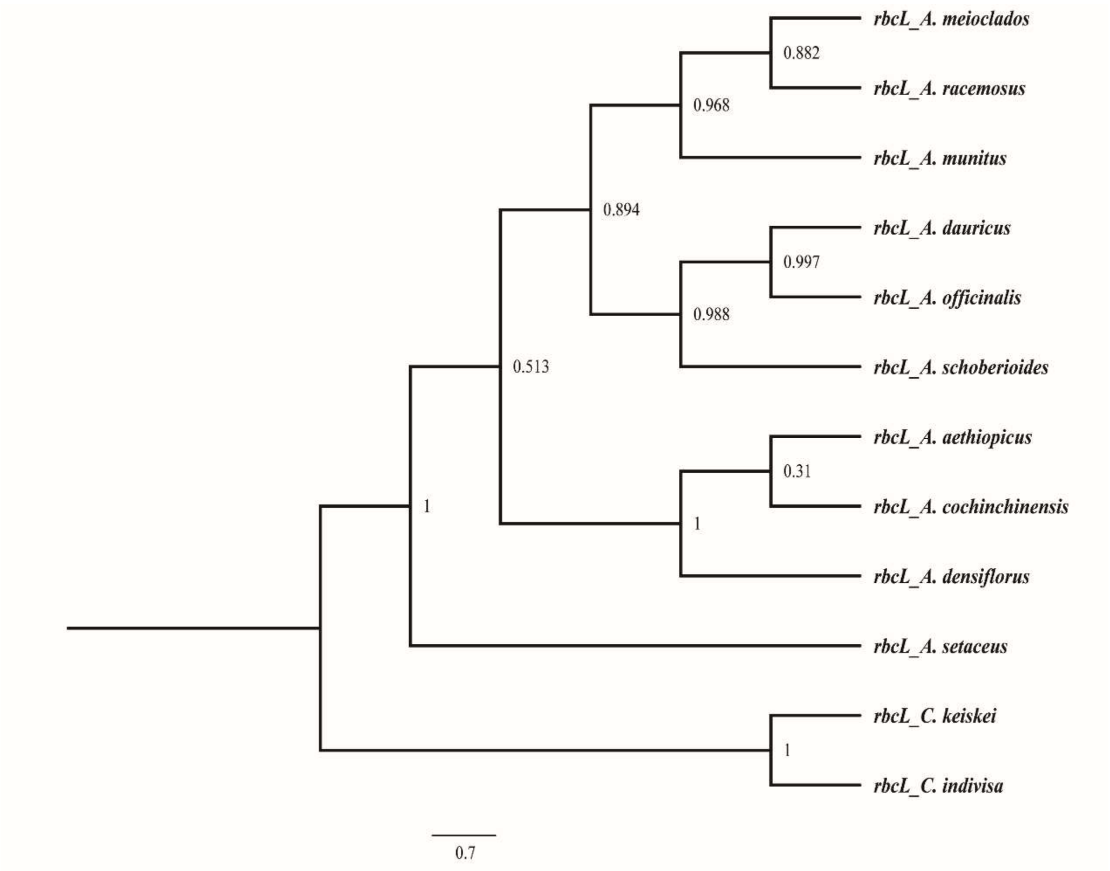

Supplementary figure 5. Neighbor Joining（NJ）tree of *Asparagus* based on *rpl32-trnL-UAG*, numbers represent Bootstrap values.

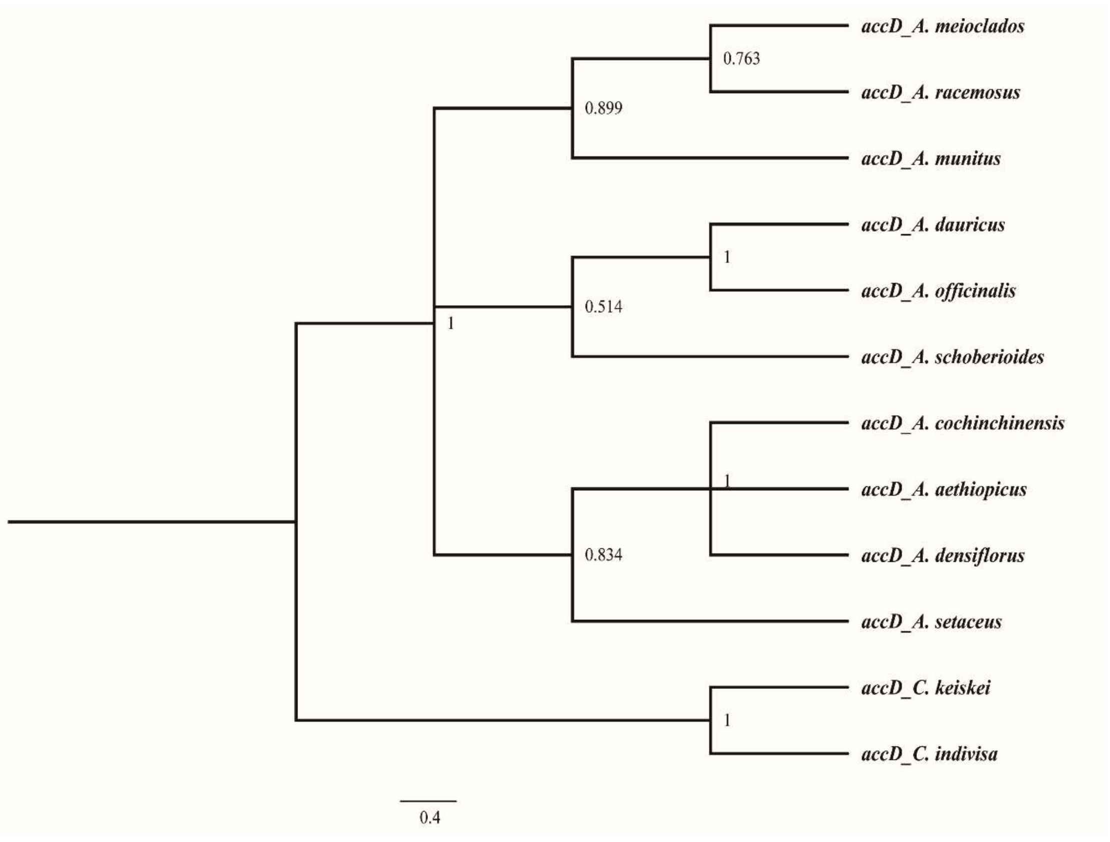

Supplementary figure 6. Neighbor Joining（NJ）tree of *Asparagus* based on *rbcL*, numbers represent Bootstrap values.
